# Simultaneous determination of moisture, 1,2-propanediol, glycerol and menthol in tobacco products using GC-TCD&FID in parallel method

**DOI:** 10.1038/s41598-025-09092-x

**Published:** 2025-07-09

**Authors:** Yaping Ma, Qinlin Xiao, Li Li, Juan Yang, Jing Wen, Yuyang Deng, Jia Guo, Xixiang Zhang, Wu Wen, Yi Shen

**Affiliations:** 1 Technology Center, China Tobacco Sichuan Industrial Co., Ltd., Chengdu, 610066 China; 2 Harmful Components and Tar Reduction in Tobacco Sichuan Key Laboratory, Chengdu, 610066 China

**Keywords:** Reconstituted tobacco leaves, GC-TCD&FID method, Glycerol, 1,2-Propanediol, Menthol, Water, Chemistry, Engineering, Chemical biology

## Abstract

Moisture, 1,2-propanediol, and glycerol are crucial quality indicators in the production and application of novel tobacco products and reconstituted tobacco leaves. However, accurately measuring these components simultaneously remains a challenge. In this study, a method applying GC-TCD&FID method for the simultaneous determination of moisture, 1,2-propanediol, glycerol and menthol was developed. The results showed that: (1) all four components exhibited good linearity, with correlation coefficients (R^2^) above 0.995 for water and above 0.999 for three alcohols. Recoveries ranged from 94.49 to 102.83%, and repeatability RSD ranged from 1.25 to 2.33%. The detection limit for water was 0.32 mg/mL, and the alcohols ranged from 0.04 to 0.21 mg/mL. (2) Sample pretreatment and GC analysis were combined into a single step, reducing reagent use, workload, and total analysis time by about 50%. Anhydrous ethanol replaced methanol as the solvent, aligning the method with green and low-carbon principles. This approach has been validated and successfully applied in production over the past 3 years, demonstrating its reliability and practicality.

## Introduction

**Fig. 1:**
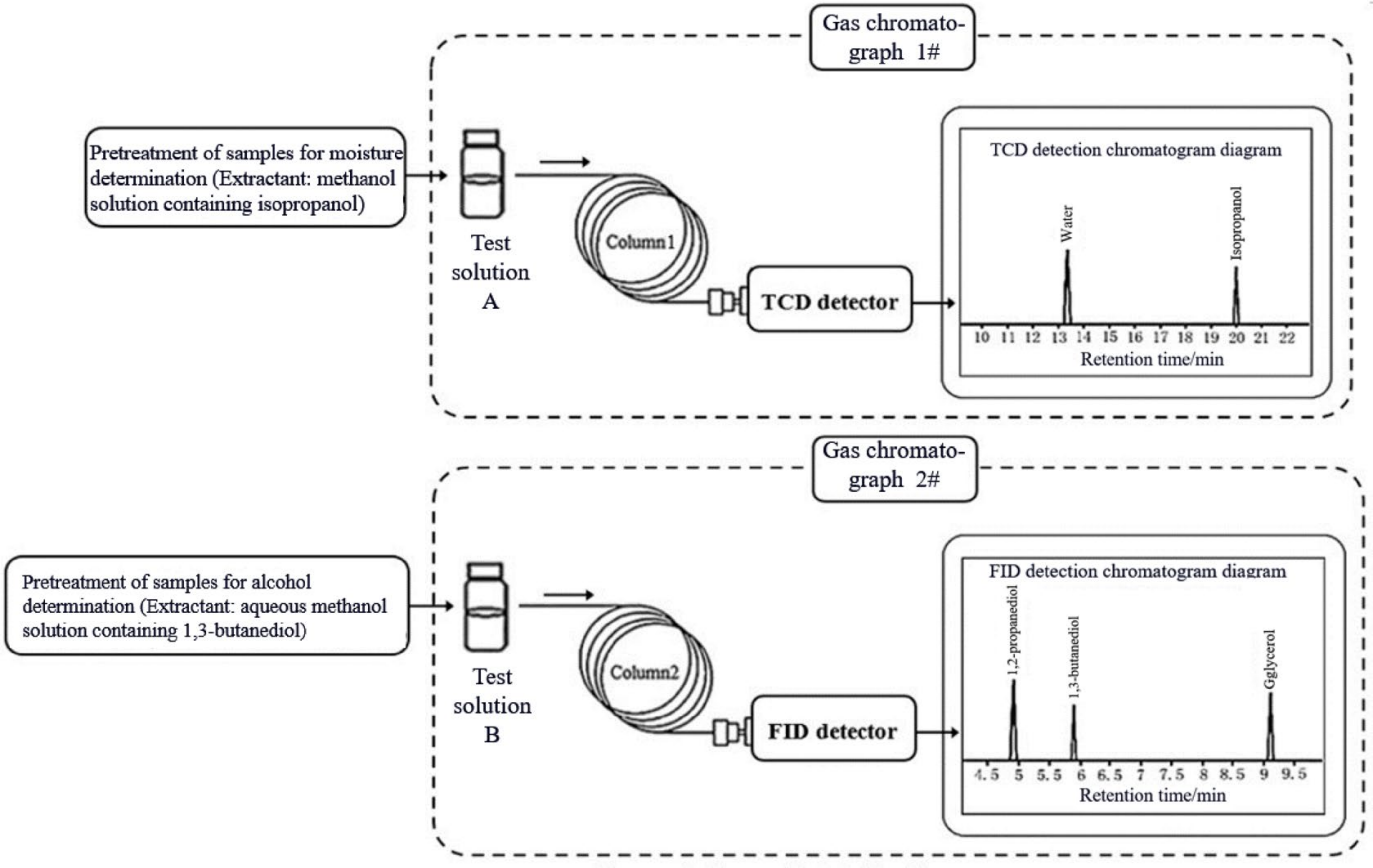
Structure of CRM57, YC/T 345—2010 and CRM60, YC/T 243—2008.

Maintaining moisture content at a certain level has a significant impact on the storage, processing durability, and sensory comfort during smoking of tobacco and tobacco products. 1,2-Propanediol and glycerol are commonly used humectants in tobacco and tobacco products, playing a crucial role in retaining moisture content^1^, extending the shelf life of tobacco products^2^ and also influencing other properties of these products. Menthol (also known as mint camphor) is a cyclic monoterpene alcohol. It is estimated that one quarter of cigarettes sold contains menthol^3,4^. The “coolness” of the smoke of menthol is viewed as pleasurable to the smokers who prefer mentholated cigarettes^5^. For instance, the application of glycerol has a notable effect on the thermogravimetric and heat release characteristics of cigar tobacco leaves, and it can promote the release of alkaline aroma components in cigar tobacco leaves under heating conditions^6^. For reconstituted tobacco sheets used in heated tobacco products, glycerol also serves as an aerosol former, and its addition correlates with the release of volatile and semi-volatile aroma components^7,8^. Meanwhile, there is an interactive relationship between the adsorption of glycerol in reconstituted tobacco sheets and their moisture content^9^. The moisture content of the tobacco core material affects the release of total particulate matter, aerosol former, and nicotine, which are the main components of heated tobacco aerosols^10^. Therefore, moisture, 1,2-propanediol, glycerol and menthol are important quality indicators that often need to be simultaneously assessed in tobacco and tobacco products. Due to the extensive application of polyhydroxy alcohols, methods for qualitative and quantitative determination of polyhydroxy alcohols via GC and its coupled techniques have been established, which are applicable to various matrices such as inks^11^, hand sanitizers^12^, water-based coatings^13^, and Chinese liquor^14^, among others. Several studies have also employed GC–MS to quantify the content of volatile organic compounds in tobacco^15,16^. However, GC–MS is not suitable for the quantitative detection of moisture. This is because the molecular weight of water (H_2_O, m/z 18) falls within the low-mass range of the mass spectrum, making it susceptible to background interference. Additionally, in electron ionization (EI) sources, water tends to fragment into H⁺ or OH⁺, resulting in weak and nonspecific signals. In the tobacco industry, the oven-drying method^17^ is commonly employed for moisture determination; however, in the presence of both moisture and alcohols, the interference caused by alcohols may adversely affect the accuracy of moisture measurement. Methods recommended by the Centre of Research Excellence on Tobacco Science (CORESTA), namely CRM57^18^ and YC/T 345-2010^19^, utilize GC-TCD method to determine moisture content in tobacco and tobacco products, while CRM60^20^ and YC/T 243-2008^21^ employ GC-FID method to measure 1,2-propanediol and glycerol. Schematic diagrams of CRM57, YC/T 345—2010 and CRM60, YC/T 243—2008 are shown in Fig. 1.

Currently, neither CORESTA nor the industry possesses a standardized method for the simultaneous determination of moisture, 1,2-propanediol, and glycerol in a single test solution for routine testing and research. To ascertain the contents of these compounds in tobacco and tobacco products, two separate sample pretreatments and two GC analyses are typically required. For instance, Zhao Meili et al.^22^ utilized YC/T 345—2010^19^ and YC/T 243—2008^21^ to measure moisture and glycerol content, respectively, during their study on the migration characteristics of moisture and glycerol in tobacco cut filler during airflow drying. Similarly, Lu Lehua et al.^23^ employed CRM57^18^ and CRM60^20^ to determine moisture content and glycerol mass fraction, respectively, when investigating the release performance of major components in reconstituted tobacco leaf for electrically heated cigarettes processed through different techniques.

**Fig. 2:**
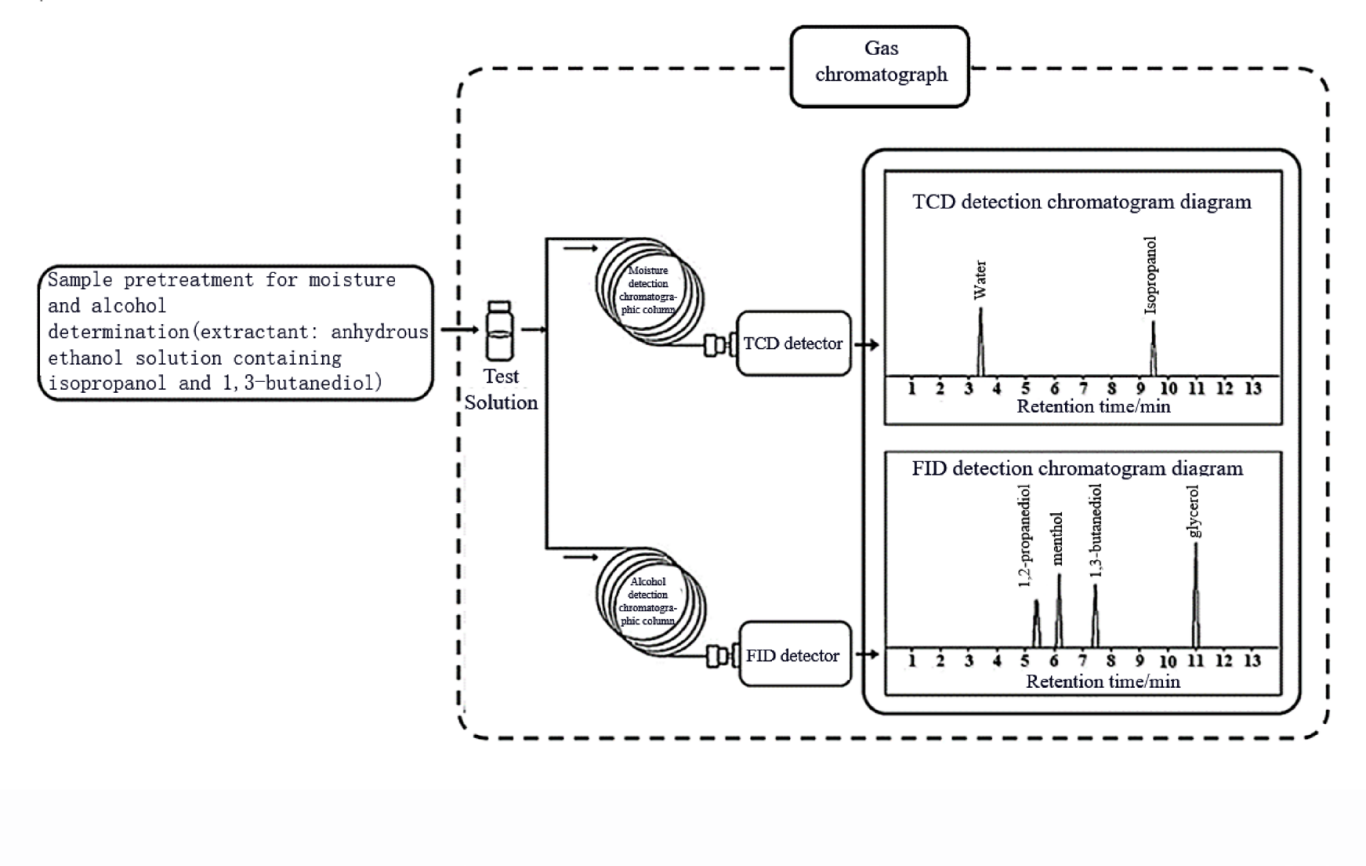
Schematic diagram of the established GC-TCD&FID Method.

This study established a method applicable to different types of tobacco and tobacco materials, which can simultaneously determine moisture, 1,2-propanediol, glycerol, and menthol. A schematic diagram of this method is shown in Fig. 2. This method achieves simultaneous injection and determination of moisture and alcohols by constructing a GC instrument equipped with two injection towers, two chromatographic columns, and two detectors (TCD and FID). Compared to the separate determination of moisture, 1,2-propanediol, and glycerol using CRM57, YC/T 345—2010 and CRM60, YC/T 243—2008, the established method requires only once sample pre-treatment and once GC analysis. The method reduces the consumption of chemical reagents and experimental consumables by approximately 50%, and reduces the sample pre-treatment time and GC analysis time by approximately 50%. In addition, anhydrous ethanol was used as the solvent instead of methanol, meeting the requirements of green, low-carbon, and sustainable development. This method will provide a suitable approach for routine quality control testing and related research work on moisture, 1,2-propanediol, glycerol, and menthol in tobacco and tobacco products.

## Materials and methods

### Materials, reagents, and instruments

Novel reconstituted tobacco leaf for heated cigarettes and conventional reconstituted tobacco leaf for cigarettes were provided by Sichuan China Tobacco Industry Co., Ltd.

Anhydrous ethanol (99.9%, ACS/HPLC grade), glycerol (99%), 1,2-propanediol (99.5%, ACS grade), isopropanol (99.5%, super dry solvent) (Beijing Hwaki Chemical Co., Ltd.); menthol (99%), 1,3-butanediol (99.5%) (Sigma–Aldrich, USA); ultrapure water (self-made, resistivity ≥ 18.2 MΩ·cm); 0.45 μm organic phase filter membranes (Tianjin Jinteng Experimental Equipment Co., Ltd.); helium (purity ≥ 99.999%, V/V) (Sichuan Messer Gas Products Co., Ltd.).

An 8890GC gas chromatograph equipped with TCD and FID detectors, HP-PLOT Q GC column (30 m × 0.53 mm × 40 µm), HP-INNOWax GC column (30 m × 0.25 mm × 0.25 µm) (Agilent, USA); AX504 electronic balance (sensitivity 0.0001 g) (Mettler Toledo, Switzerland); ZD-88 full-temperature air bath oscillator (Shanghai Precision Instrumentation Co., Ltd.); Milli-Q ultrapure water system (Millipore, USA); 8510E-DTH ultrasonic cleaner (Branson Ultrasonics, USA).

### Methods

#### Preparation of internal standard stock solution

Accurately weigh 5 g of each isopropanol and 1,3-butanediol, and dilute to 50 mL with anhydrous ethanol to prepare a 100 mg/mL internal standard stock solution.

#### Preparation of extraction solvent

Precisely measure 30 mL of the internal standard stock solution and dilute to 1000 mL with anhydrous ethanol to prepare a 3 mg/mL extraction solvent.

#### Preparation of moisture standard working solutions

Accurately weigh 0.5 g of water and dilute to 50 mL with anhydrous ethanol to prepare a 10 mg/mL moisture standard stock solution. Precisely measure 0, 0.2, 0.5, 1.0, 2.0, 4.0, 8.0 mL of the moisture standard stock solution into different 10 mL volumetric flasks, then accurately add 0.3 mL of the internal standard stock solution to each, and dilute to volume with anhydrous ethanol. The resulting solutions have moisture concentrations of 0, 0.2, 0.5, 1.0, 2.0, 4.0, 8.0 mg/mL, respectively, with an internal standard concentration of 3 mg/mL in each.

#### Preparation of 1,2-propanediol, glycerol, and menthol standard working solutions

Accurately weigh 0.2 g of 1,2-propanediol, 1.0 g of glycerol, and 0.2 g of menthol, and dilute to 50 mL with anhydrous ethanol to prepare a mixed standard stock solution with concentrations of 4, 20, and 4 mg/mL, respectively. Precisely measure 0.05, 0.2, 0.5, 1.0, 4.0, 8.0 mL of the standard stock solution into different 10 mL volumetric flasks, then accurately add 0.3 mL of the internal standard stock solution to each, and dilute to volume with anhydrous ethanol. The resulting solutions have 1,2-propanediol and menthol concentrations of 0.02, 0.08, 0.2, 0.4, 1.6, 3.2 mg/mL, respectively, glycerol concentrations of 0.1, 0.4, 1.0, 2.0, 8.0, 16.0 mg/mL, respectively, with an internal standard concentration of 3 mg/mL in each.

#### Tobacco sample preparation

Sheet sample pretreatment: Randomly select 100 g of the sample to be tested, cut it into fragments of approximately 0.4 cm × 0.4 cm, thoroughly mix, and store in a sealed, light-protected container for later use.

Filament sample pretreatment: Randomly select 100 g of the sample to be tested, thoroughly mix, and store in a sealed, light-protected container for later use.

Preparation of test solution: Weigh 1.5 g of the sample (to 0.001 g precision) into a 50 mL stoppered conical flask, accurately add 25 mL of the extraction solvent, and oscillate and extract at 30 °C and 160 r/min for 3 hours^18^. Filter the supernatant, and the filtrate is the test solution.

Preparation of blank solution: Prepare a blank solution without adding the sample, following the same procedure as for the test solution, for the determination of blank moisture value. Due to the solvent’s absorption of water, the blank moisture value should be subtracted when calculating the moisture content of the test solution.

Unless otherwise specified in the following experiments, each sample was determined in triplicate, and the average value was taken.

### GC-TCD&FID analysis

Moisture determination conditions: HP-PLOT Q GC column (30 m × 0.53 mm × 40 µm); TCD detector temperature: 270 °C; injection port temperature: 260 °C; split injection mode with a split ratio of 10:1; injection volume: 1.0 μL; carrier gas: helium (≥ 99.999%), constant flow mode, flow rate: 6.0 mL/min; make-up gas: helium, flow rate: 10 mL/min; reference flow rate: 25 mL/min.

1,2-propanediol, glycerol, and menthol determination conditions: HP-INNOWax GC column (30 m × 0.25 mm × 0.25 µm); FID detector temperature: 270 °C; injection port temperature: 260 °C; split injection mode with a split ratio of 25:1; injection volume: 1.0 µL; carrier gas: helium (≥ 99.999%), constant flow mode: flow rate set at 1.2 mL/min; air flow rate at 400 mL/min; hydrogen flow rate at 40 mL/min; make-up gas is helium with a flow rate of 25 mL/min.

Heating Procedure: initiate at a temperature of 110 °C and maintain for 1 min. Subsequently, increase the temperature at a rate of 10 °C/min to reach 150 °C, where it is held for 2 min. Further elevate the temperature at a rate of 20 °C/min to 240 °C and maintain for 5 min.

## Results and discussion

### Selection of sample morphology

For flake samples, the influence of two sample morphologies (4 mm × 4 mm cuboid and powdered) on the extraction efficiency of target compounds using methanol as the extraction solvent under oscillatory extraction conditions was investigated. The results (Table 1) indicate that the extraction efficiency for 1,2-propanediol is approximately 6% higher in the cuboid form compared to the powdered form; for menthol, the extraction efficiency is approximately 9% higher in the cuboid form; however, for glycerol, the extraction efficiency is approximately 2% lower in the cuboid form. This may be because 1,2-propanediol has a boiling point of 187 °C, and menthol has a boiling point of 216 °C. During the pulverization process of tobacco samples, friction generates heat, causing more volatilization of 1,2-propanediol and menthol, which have lower boiling points, resulting in higher extraction efficiency of 1,2-propanediol and menthol from cuboid tobacco samples compared to powdered samples. In contrast, glycerol has a boiling point of 290 °C and is difficult to volatilize, making powdered tobacco samples more favorable for its extraction. The extraction efficiency for moisture is comparable between the cuboid and powdered forms. Taking these considerations into account, the 4 mm × 4 mm cuboid morphology was selected as the sample morphology for flake samples.

**Table 1:** Effects of sample morphology on extraction efficiency of target compounds (n = 3).

Sample No	Sample morphology	Water content (mg/mL)	1,2-propanediol content (mg/mL)	Glycerol content (mg/mL)	Menthol content (mg/mL)
1 #	4 mm × 4 mm cuboid	62.6	27.5	178.2	13.0
	Powdered	62.9	25.8	181.6	11.8
2 #	4 mm × 4 mm cuboid	64.3	35.9	172.1	21.1
	Powdered	64.7	33.7	174.8	19.2

### Selection of extraction solvent

The extraction efficiencies of target compounds using isopropanol, anhydrous ethanol, and methanol were investigated. The results (Table 2) indicate that anhydrous ethanol exhibits significantly higher extraction efficiency for glycerol compared to methanol and isopropanol. Furthermore, anhydrous ethanol demonstrates slightly higher extraction efficiencies for water, 1,2-propanediol, and menthol than methanol and isopropanol. Therefore, anhydrous ethanol was selected as the extraction solvent.

**Table 2:** Effects of extraction solvent on extraction efficiency of target compounds (n = 3).

Sample No	Extraction solvent	Water content (mg/mL)	1,2-propanediol content (mg/mL)	Glycerol content (mg/mL)	Menthol content (mg/mL)
1#	Isopropanol	62.1	26.9	151.6	12.4
	Anhydrous ethanol	62.6	27.5	178.2	13.0
	Methanol	62.3	27.1	171.1	12.7
2#	Isopropanol	63.8	35.4	148.2	20.5
	Anhydrous ethanol	64.3	35.9	172.1	21.1
	Methanol	63.6	35.5	166.2	20.7

### Comparative determination of alcoholic compounds in the same standard solution using FID and TCD detectors

Kang et al.^24^ established a GC-TCD method for the simultaneous detection of water, nicotine, 1,2-propanediol, glycerol, triacetin, and menthol in aerosol from heat-not-burn cigarettes. Ye et al.^25^ also developed a GC-TCD method for the simultaneous determination of water, 1,2-propanediol, nicotine, and glycerol in tobacco materials for heat-not-burn cigarettes. To assess the feasibility of using the TCD detector for routine quality control measurements of 1,2-propanediol and glycerol in tobacco and tobacco products, standard working solutions of 1,2-propanediol, glycerol, and menthol were determined using both the established method and the literature method^24,25^. The results (Table 3) show that the detection values (peak areas) of the TCD detector for 1,2-propanediol, glycerol, menthol, and 1,3-butanediol are significantly lower than those of the FID detector. The results indicate that the FID exhibits higher detection sensitivity towards these four alcohols, rendering it more suitable for routine quality control testing.

**Table 3:** Determination of alcohols in the same standard solution by FID and TCD detector.

Target compound	Concentration (mg/mL)	FID detection value (Peak area)	TCD detection value (Peak area)	Peak area ratio (FID/TCD)
1,2-Propanediol	3.432	764.2	54.4	14.1
Glycerol	16.354	2785.3	211.6	13.2
Menthol	3.291	1621.6	35.6	45.7
1,3-Butanediol	3.013	794.9	46.2	17.2

### Methodological validation

Reference was made to the "Guiding Principles for Analytical Method Validation" numbered 9101 in the "General Technical Requirements" section of the 2020 edition, Part IV, of the "Pharmacopoeia of the People’s Republic of China"^26^ to investigate the linearity, detection limit, quantitation limit, recovery, reproducibility, robustness and selectivity of the method.

#### Linearity, detection limit, and quantitation limit

The concentration ranges of the compounds in the standard working solutions were set to basically cover the content ranges of the target compounds commonly found in tobacco and tobacco products. The detection limit and quantitation limit were determined using the signal-to-noise ratio method: by comparing the signals measured from known low-concentration samples with the signals measured from blank samples, the contents of the corresponding target compounds at signal-to-noise ratios of 3:1 and 10:1 were used to determine the detection limit and quantitation limit, respectively.

Linearity assessment of the water determination method: Seven levels of water standard working solutions were measured according to the water determination conditions to establish a water standard curve (with the ratio of water concentration to isopropanol concentration as the abscissa and the ratio of water peak area to isopropanol peak area as the ordinate). The linearity of the standard curve was evaluated using the correlation coefficient.

Linearity assessment of the determination methods for 1,2-propanediol, glycerol, and menthol: Six levels of standard working solutions were measured according to the determination conditions for 1,2-propanediol, glycerol, and menthol, and standard curves for 1,2-propanediol, glycerol, and menthol were established respectively (with the ratio of target compound concentration to internal standard concentration as the abscissa and the ratio of target compound peak area to internal standard peak area as the ordinate). The linearity of the standard curves was evaluated using the correlation coefficient.

The results (Table 4) indicate that the standard curves for the four target compounds exhibit good linearity, with low detection limits and quantitation limits, and high method sensitivity. The recovery rates for the three alcohols range from 94.49 to 102.30%, with an RSD of 1.25–2.32%. Additionally, the linearity of the standard curves mentioned above surpasses the research findings of Wang, L. Q., et al., regarding the determination of alcohols^27^. This method exhibits a significant enhancement in both the detection range and precision for quantifying 1,2-propanediol and glycerol compared to the method proposed by Adrielle, X. C.^28^.

**Table 4:** Linear parameters, detection limits, and quantitation limits of four target compounds.

Target compound	Linear range (mg/mL)	Regression equation	Correlation coefficient	Detection limit (mg/mL)	Quantitation limit (mg/mL)
Water	0.2–9.0	y = 15.671x + 5.205	0.9987	0.32	0.97
1,2-Propanediol	0.02–3.2	y = 223.927x − 1.643	0.9999	0.21	0.58
Glycerol	0.1–16.0	y = 171.310x − 7.484	0.9999	0.15	0.67
Menthol	0.02–3.2	y = 495.415x − 0.612	0.9999	0.04	0.21

#### Recovery and reproducibility of the method

Recovery assessment method: A known amount of a standard compound of known purity was precisely added to a sample with a known content (such that the sum of the added amount and the original content remained within the linear range of the standard curve). The sample was then measured according to the established method. The recovery was calculated by dividing the difference between the measured value and the original content in the sample by the added amount of the standard compound. Seven parallel measurements were performed.

The results (Table 5) demonstrate that the method exhibits high accuracy and excellent reproducibility.

**Table 5:** Recovery and precision of the method (n = 7).

Target compound	Original content/mg	Spike amount/mg	Measured range/mg	Recovery range/%	RSD/%
Water	93.9	70.6	67.9–72.6	96.13–102.83	2.33
1,2-Propanediol	41.25	30.3	29.3–31.0	96.80–102.30	2.32
Glycerol	267.3	200.2	191.6–198.6	95.69–99.22	1.25
Menthol	19.5	14.1	13.3–13.9	94.49–98.33	1.36

#### Robustness of the method

During the development and validation process of this method, the robustness was evaluated. Deliberate minor variations were introduced to parameters such as the carrier gas flow rate, injector temperature, temperature programming of GC column, and detector temperature during GC determination to investigate whether these changes would affect the measurement results. The findings indicated that the measurement results remained consistent despite these intentional minor parameter variations, demonstrating the reliability of the method under normal usage conditions. The parameter variation ranges during the robustness evaluation are presented in Table 6.

**Table 6:** Variation ranges of relevant parameters during the evaluation of method robustness.

Parameters	Parameter set value	Parameter variation range during robustness evaluation
Parameters for moisture determination		
Injector temperature	260 °C	257–263 °C
Carrier gas flow rate	6.0 mL/min	5.8–6.2 mL/min
Heating procedure of the GC column	Initial temperature: 110 °C, held for 1 min; then increased to 150 °C at a rate of 10 °C/min and held for 2 min; finally increased to 240 °C at a rate of 20 °C/min and held for 5 min	Initial temperature: 108–112 °C, held for 1 min; then increased to 148–152 °C at a rate of 9.5–10.5 °C/min and held for 2 min; finally increased to 238–242 °C at a rate of 19.5–20.5 °C/min and held for 5 min
TCD temperature	270 °C	267–273 °C
Parameters for the determination of 1,2-propanediol, glycerol, and menthol		
Injector temperature	260 ℃	257–263 °C
Carrier gas flow rate	1.2 mL/min	1.1–1.3 mL/min
Heating procedure of the GC column	Initial temperature: 110 °C, held for 1 min; then increased to 150 °C at a rate of 10 °C/min and held for 2 min; finally increased to 240 °C at a rate of 20 °C/min and held for 5 min	Initial temperature: 108–112 °C, held for 1 min; then increased to 148–152 °C at a rate of 9.5–10.5 °C/min and held for 2 min; finally increased to 238–242 °C at a rate of 19.5–20.5 °C/min and
FID temperature	270 °C	267–273 °C

As moisture, 1,2-propanediol, glycerol, and menthol are simultaneously determined on the same GC, and the two columns share a common column oven. Therefore, the heating procedure for determining moisture and alcohol compounds is the same.

#### Selectivity

**Fig. 3:**
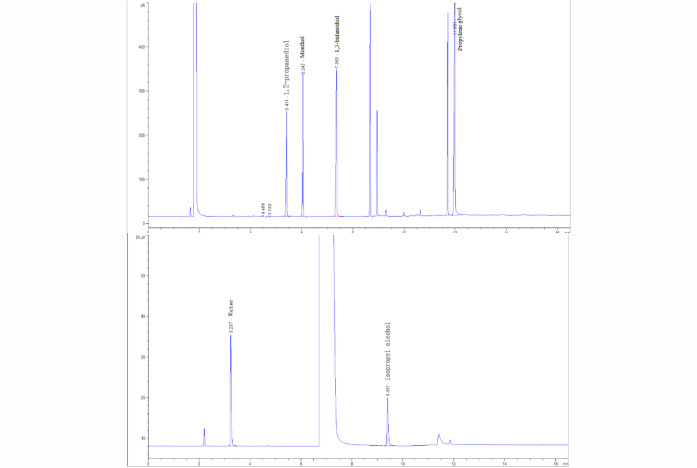
Gas chromatogram for water 1,2-propanediol, glycerol, and menthol detection in tobacco materials.

Figure 3 displays the GC obtained from analyzing tobacco material samples using the established method. As can be observed from the figure, the chromatographic peaks of the target compounds 1,2-propanediol, glycerol, menthol, and the internal standard compound 1,3-butanediol, as well as those of their adjacent compounds, achieved complete baseline separation. Similarly, the chromatographic peaks of the target compound water and the internal standard isopropanol, along with those of their adjacent compounds, also achieved complete baseline separation. This indicates that the method exhibits good selectivity.

### Sample analysis

The tobacco material samples were analyzed according to the established method. The gas chromatogram for water, 1,2-propanediol, glycerol, and menthol determination is presented in Fig. 3. The results indicate that the peaks of each target compound and internal standard are sharp and well-separated.

**Fig. 4:**
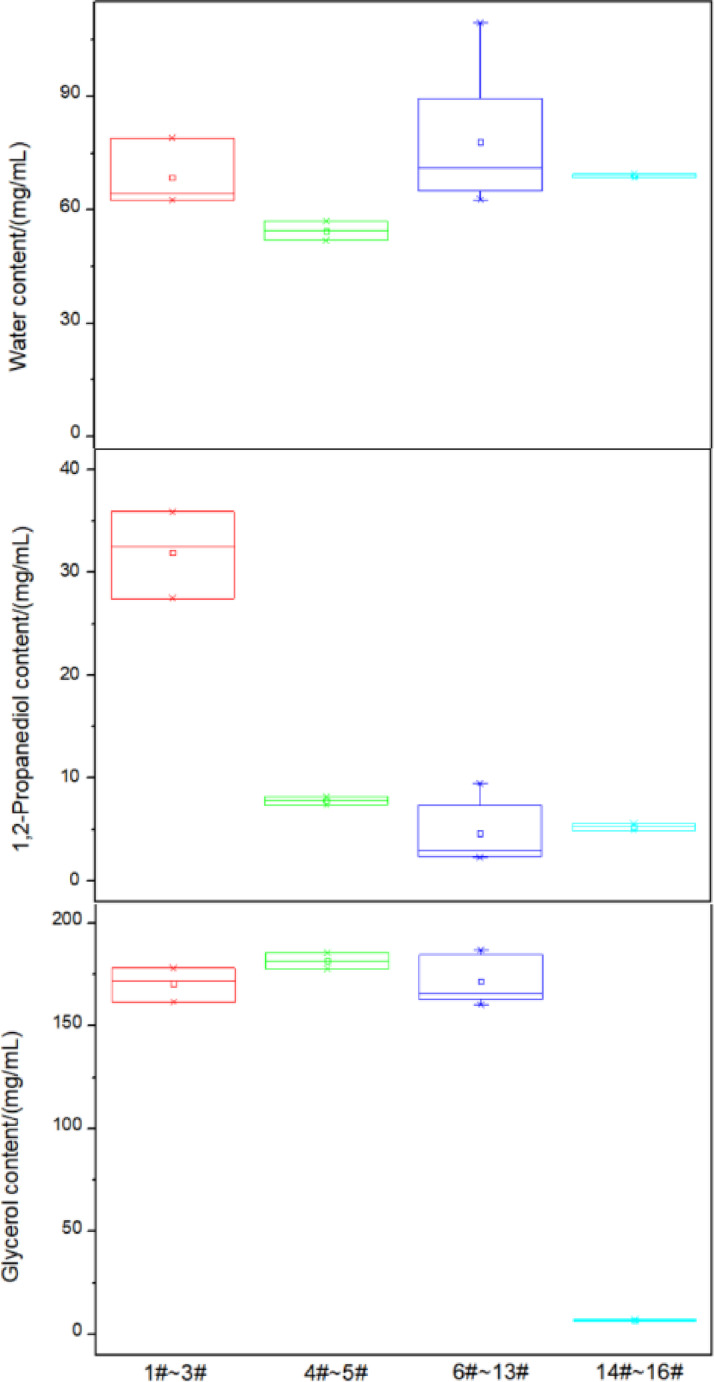
Boxplots of water,1,2-propanediol, glycerol for four types of tobacco products. Error bars at the top of each column indicate the standard deviation of the measurement.

The measurement results for the 16 tobacco material samples are presented in Table 7. Specifically, samples numbered 1#–3# represent, 4#–5# for sheet-type heated tobacco products (produced via dry papermaking), 6#–13# for filament-type heated tobacco products (manufactured through the thick slurry method), and 14#–16# for filament-type conventional cigarettes. This is shown in Fig. 4, the average moisture content follows the order: reconstituted tobacco leaves for filament-type heated tobacco products (thick slurry method) > reconstituted tobacco leaves for filament-type conventional cigarettes > reconstituted tobacco leaves for sheet-type mentholated heated tobacco products > reconstituted tobacco leaves for sheet-type heated tobacco products (dry papermaking). The average 1,2-propanediol content in reconstituted tobacco leaves for sheet-type mentholated heated tobacco products is significantly higher than that in other sample types. The average glycerol content in reconstituted tobacco leaves for filament-type conventional cigarettes is considerably lower than in other types. Notably, there is considerable variation in the menthol content among different samples of reconstituted tobacco leaves for sheet-type mentholated heated tobacco products. The component content variations are relatively small for both reconstituted tobacco leaves for sheet-type heated tobacco products (dry papermaking) and filament-type conventional cigarettes, with coefficient of variation (CV) values ranging from 0.8 to 7.2%. The CVs for glycerol content across all four types of tobacco material samples are also low, falling within the range of 3.1–6.7%. However, the CVs for moisture and 1,2-propanediol content in reconstituted tobacco leaves for filament-type heated tobacco products (thick slurry method) are relatively high, at 23.5% and 63.9%, respectively.

**Table 7:** Analysis results of 16 tobacco materials.

No	Sample state	Type	Water content (mg/mL)	1,2-Propanediol content (mg/mL)	Glycerol content (mg/mL)	Menthol content (mg/mL)
1#	Sheet-like	Reconstituted tobacco leaf for menthol Heated tobacco products	62.6	27.5	178.2	13.0
2#			64.3	35.9	172.0	21.0
3#			79.0	32.5	161.7	17.3
4#		Reconstituted tobacco leaf for heated tobacco products (Dry papermaking)	51.9	8.2	177.7	–
5#			57.0	7.4	185.7	–
6#	Filamentous	Reconstituted tobacco leaf for heated tobacco products (Dense slurry method)	62.7	7.7	185.1	–
7#			75.4	7.1	184.2	–
8#			109.6	3.4	167.5	–
9#			103.7	2.3	164.7	–
10#			69.3	2.4	164.1	–
11#			67.2	2.3	160.4	–
12#			62.9	2.4	161.8	–
13#			72.8	9.5	187.0	–
14#		Reconstituted tobacco leaf for traditional cigarettes	69.2	5.6	6.7	–
15#			69.6	5.3	7.1	–
16#			68.5	4.9	6.5	–

### Establishment of quality inspection technical standards for Enterprises

Using the established method, quality inspection technical standards were formulated by Sichuan China Tobacco Industrial Co., Ltd. for routine quality control testing of water, 1,2-propanediol, glycerol, and menthol in various tobacco and tobacco products. The standards have been in operation for three years with satisfactory results.

From January 2022 to the present, this quality inspection technical standard has been employed for quarterly quality supervision and inspection of the reconstituted tobacco leaf for heated tobacco products used by the company. Approximately 68 batches of samples are analyzed each year. Up to now, more than 200 batches of samples have been subjected to quality supervision and inspection using this standard method. The relevant performance indicators of the reconstituted tobacco leaf for heated tobacco products are shown in Table 8, and the partial test data are presented in Table 9. According to feedback from the company’s quality control team, the use of this standard method has played a crucial role in promoting the quality stability of the reconstituted tobacco leaf samples.

**Table 8:** Performance indicators related to reconstituted tobacco.

No	Performance indicators’s content (mg/mL)	Technical requirements	
		Reconstituted tobacco leaf for heated tobacco products (dense slurry method)	Reconstituted tobacco leaf for heated tobacco products (dry papermaking)
1	Moisture	60.0 ± 15.0	90.0 ± 17.0
2	Glycerol	185.0 ± 20.0	195.0 ± 20.0
3	1,2-Propanediol	30.0 ± 7.0	25.0 ± 7.0
4	Menthol	18.0 ± 5.0	18.0 ± 5.0

Note: The technical requirements for menthol are only applicable to reconstituted tobacco samples with menthol added.

**Table 9:** The partial test data of reconstituted tobacco leaf samples.

No	Types of reconstituted tobacco leaf samples	Water content (mg/mL)	Glycerol content (mg/mL)	1,2-Propanediol content (mg/mL)	Menthol content (mg/mL)
1*	Dense slurry method	55.6	173.5	19.3	0.00
2*	Dense slurry method	52.1	177.3	18.9	0.00
3*	Dense slurry method	68.1	166.9	20.1	0.00
4*	Dense slurry method	62.4	179.5	21.3	0.00
5*	Dense slurry method	72.8	174.6	21.8	0.00
6*	Dense slurry method	63.5	173.7	30.9	0.00
7*	Dense slurry method	73.7	167.3	20.7	0.00
8*	Dense slurry method	66.1	168.1	28.2	0.00
9*	Dense slurry method	67.3	181.2	19.9	0.00
10*	Dense slurry method	59.7	182.7	23.5	0.00
11*	Dense slurry method	73.3	190.1	19.2	0.00
12*	Dense slurry method with menthol	59.7	167.5	26.9	1.93
13*	Dense slurry method with menthol	53.5	165.9	29.1	1.56
14*	Dense slurry method with menthol	62.9	169.2	30.6	1.90
15*	Dry papermaking	76.3	183.4	24.8	0.00
16*	Dry papermaking	81.6	189.6	25.1	0.00

## Conclusion

A GC-TCD&FID method for the simultaneous determination of moisture, 1,2-propanediol, glycerol, and menthol contents in tobacco materials was established. (1) The standard curves of the four components exhibited good linearity (R^2^ > 0.995 for moisture and R^2^ > 0.999 for three alcohols), high accuracy (recovery rates of 94.49–102.83%), good reproducibility (RSD of 1.25–2.33%), and high sensitivity (limits of detection for water was 0.32 mg/mL and limits of detection for the three alcohols ranging from 0.04 to 0.21 mg/mL). (2) The developed method streamlines the original workflow, which involves the process of sample pretreatment and GC analysis has been consolidated from two separate steps into one. Thereby the workload, reagent consumption , time required for both sample pretreatment and GC analysis were reduced by about 50%. And anhydrous ethanol was used as the solvent instead of methanol, meeting the requirements for green, low‒carbon, and sustainable development. (3) The established method has been in operation for three years in the quality inspection technical standards of related products at China Tobacco Sichuan Industrial Co., Ltd., with good results.

## Data Availability

The data for this study will be made available upon request from the corresponding author.
